# Progression of C-reactive protein from birth through preadolescence varies by mode of delivery

**DOI:** 10.3389/fped.2023.1155852

**Published:** 2023-06-14

**Authors:** Alexandra R. Sitarik, Christine C. Johnson, Albert M. Levin, Susan V. Lynch, Dennis R. Ownby, Andrew G. Rundle, Jennifer K. Straughen, Ganesa Wegienka, Kimberley J. Woodcroft, Andrea E. Cassidy-Bushrow

**Affiliations:** ^1^ Department of Public Health Sciences, Henry Ford Health, Detroit, MI, United States; ^2^ Division of Gastroenterology, Department of Medicine, University of California, San Francisco, CA, United States; ^3^ Division of Allergy and Clinical Immunology, Department of Pediatrics, Medical College of Georgia at Augusta University, Augusta, GA, United States; ^4^ Department of Epidemiology, Mailman School of Public Health, Columbia University, New York, NY, United States

**Keywords:** caesarean section, inflammation, high-sensitivity C-reactive protein, birth cohort, developmental origins of health and disease

## Abstract

**Introduction:**

Delivery via caesarean section (C-section) has been associated with an increased risk of childhood chronic diseases such as obesity and asthma, which may be due to underlying systemic inflammation. However, the impact of specific C-section types may be differential, as emergency C-sections typically involve partial labor and/or membrane rupture. Our objectives were to determine if mode of delivery associates with longitudinal profiles of high sensitivity CRP (hs-CRP) —a marker of systemic inflammation—from birth through preadolescence, and to examine if CRP mediates the association between mode of delivery and preadolescent body mass index (BMI).

**Methods:**

Data from the WHEALS birth cohort (*N* = 1,258) were analyzed; 564 of the 1,258 children in the cohort had data available for analysis. Longitudinal plasma samples (birth through 10-years of age) from 564 children from were assayed for hs-CRP levels. Maternal medical records were abstracted to obtain mode of delivery. Growth mixture models (GMMs) were used to determine classes of hs-CRP trajectories. Poisson regression with robust error variance was used to calculate risk ratios (RRs).

**Results:**

Two hs-CRP trajectory classes were identified: class 1 (76% of children) was characterized by low hs-CRP, while class 2 (24% of children) was characterized by high and steadily increasing hs-CRP. In multivariable models, children delivered via planned C-section had 1.15 times higher risk of being in hs-CRP class 2, compared to vaginal deliveries (*p* = 0.028), while no association was found for unplanned C-section deliveries [RR (95% CI) = 0.96 (0.84, 1.09); *p* = 0.49]. Further, the effect of planned C-section on BMI z-score at age 10 was significantly mediated by hs-CRP class (percent mediated = 43.4%).

**Conclusions:**

These findings suggest potentially beneficial effects of experiencing partial or full labor, leading to a lower trajectory of systemic inflammation throughout childhood and decreased BMI during preadolescence. These findings may have implications for chronic disease development later in life.

## 1. Introduction

Delivery via caesarean section (C-section) has been on the rise, with the rate nearly doubling worldwide between 2000 and 2015 (12.1% and 21.1% of all births, respectively) (1). Though sometimes medically necessary and life-saving, procedures can also be planned in advance upon maternal request without medical indications based on patient/provider preferences. However, C-section delivery may also have negative health consequences for offspring later during childhood, with a recent meta-analysis demonstrating an increased risk of childhood obesity, respiratory tract infections, and asthma (2). Beyond childhood, the consequences of C-section delivery has also been shown to persist up to 40 years later, with an increased risk of inflammatory diseases including diabetes, arthritis, coeliac disease, and inflammatory bowel disease (3). We and others have found that type of C-section can differentially impact these associations, with elective—but not emergent—C-section having an increased risk of childhood obesity (4) and lower respiratory tract infections (5).

The biological mechanism linking C-section delivery to adverse outcomes in children is not well understood. However, a prominent theory is that C-section delivery results in a dysbiotic infant gut microbiota due to lack of vaginal microbiota exposure during birth (6, 7); microbiological disturbances have been shown to have sustained impacts on overall health, including immune dysregulation (8) and metabolic dysfunction (9). Inflammation also plays an important role in the development of cardiometabolic diseases, with key drivers including diet (10) and chronic stress (11). C-reactive protein (CRP)—which is produced by the liver following stimulation by interleukin-6 generated by adipose tissue (12)—may be an important biomarker in this causal pathway, as it is an indicator of inflammation that has been shown to be strongly influenced by obesity (13) and increases the risk of cardiovascular disease (14) and atherosclerosis (15). While very high CRP levels are generally indicative of active infection or injury (16), moderately high values tend to reflect systemic inflammation. As we previously found an association between mode of delivery and childhood obesity (4), demonstrating an association with CRP levels in these same children would additionally indicate a high-risk inflammatory process following C-section delivery, which may have implications for future cardiovascular health as well as a multitude of other chronic diseases tied to inflammation. Our objective was to examine the association between mode of delivery and longitudinal profiles of CRP expression (measured by a high-sensitivity CRP assay, hs-CRP) from birth through preadolescence, and determine if hs-CRP mediates the association between mode of delivery and BMI at age 10. We hypothesized that children delivered via planned C-section would be more likely to have a high hs-CRP trajectory throughout preadolescence, and that hs-CRP trajectory would mediate the association between mode of delivery and BMI at age 10.

## 2. Methods

### 2.1. Study population

Maternal-child pairs from the Wayne County Health Environment Allergy and Asthma Longitudinal Study (WHEALS) birth cohort study were analyzed. Cohort details have been previously published (17). Briefly, a total of 1,258 pregnant women ages 21–49 years receiving care at Henry Ford Health obstetrics clinics in metropolitan Detroit were recruited between 2003 and 2007. Women resided in the city of Detroit or surrounding suburban areas, which resulted in a racially and socioeconomically diverse sample. Participants have been followed longitudinally with the following assessments: a prenatal questionnaire, 1-month, 6-month, and 1-year home visits, a 2-year clinic visit, a 3-year questionnaire, and a 10-year clinic visit. All mothers provided written, informed consent; children provided written, informed assent at the 10-year clinic visit. The study was approved by the institutional review board at Henry Ford Health.

### 2.2. Covariates

Mothers self-reported the following at the prenatal interview: race, insurance coverage, household income, education, marital status, previous pregnancies, smoking during pregnancy, household environmental tobacco smoke, prenatal alcohol use, indoor pets, history of asthma and allergies, and home address, which was used to define urban or suburban residence. Prenatal and delivery electronic medical records were abstracted to obtain mode of delivery (vaginal vs. C-section) and specific delivery type (vaginal vs. planned C-section vs. unplanned C-section due to dysfunctional labor, fetal malposition, fetal distress, or maternal distress). Note that the rate of missingness is higher for specific delivery type because this information could not always be found. Body mass index (BMI) at the first prenatal visit, prenatal antibiotic and antifungal use, any hypertensive disorders during pregnancy, gestational diabetes, gestational age at delivery, and birthweight were also chart abstracted. Birthweight z-scores were calculated using the US population in 1999–2000 as a reference (18). Height and weight was measured during clinic visits at age 2 and 10; BMI z-scores were calculated using the 2000 CDC growth charts (19). A complete blood count with differential was performed on each blood sample collected at the 10-year visit. Pubertal development at age 10 was quantified using the Pubertal Development Scale (20). Briefly, children were asked to self-rate their development for various characteristics related to puberty and report whether there had been no development, development had barely begun, development was definitely underway, or development was complete. These questions were used to create the following collapsed puberty categories: pre - early puberty, mid puberty, and late - post puberty. Breastfeeding was maternal-reported in the 1-month questionnaire.

### 2.3. hs-CRP measurement

Banked plasma samples collected at birth (via umbilical cord blood), 6-months, 1-year, 2-years, and 10-years were used to quantify hs-CRP levels. Children were selected for hs-CRP measurement (at all time points with a banked sample) if a 10-year plasma sample was collected. Plasma samples were stored at −80°C from time of collection. The high-sensitivity enzyme-linked immunosorbent assay (ELISA) was used to measure CRP, per manufacturer's instructions (Oxis International Inc., Foster City, CA). Concentrations of hs-CRP (mg/L) were calculated from a standard curve. Potential cases of active infection were defined as those with hs-CRP > 10 mg/L and white blood cell count >10 K/ul at age 10 (21, 22).

### 2.4. Statistical analysis

#### 2.4.1. Missing data

Because loss to follow-up can affect the internal validity of estimates, inverse probability weighting (IPW) was used to attempt to correct for this bias (23). Analytic sample inclusion was used as the outcome in a logistic regression model with a variety of baseline and early life covariates that may predict loss to follow-up (listed in Supplementary Table S1). Subject weights were calculated as the inverse probability of the “treatment” received (included vs. excluded). Covariate balance was assessed using the standardized differences before and after weighting, with imbalance defined as absolute value >0.20.

Because some missingness was present in covariates in the analytic subsample and because not all children had a hs-CRP measurement at every time point, multiple imputation was performed, which can reduce bias and increase precision compared to complete-case estimates (24). The SAS procedure *mi* with the fully conditional specification algorithm was used to generate imputed datasets. A total of 36 imputations were performed, which was selected because 36% of subjects had incomplete data. This is a commonly used rule of thumb that considers both efficiency and reproducibility (25), as a small number of imputations may not sufficiently estimate standard errors despite high relative efficiency (26).

#### 2.4.2. Causal model and covariate adjustment

In order to conceptualize our hypothesized relationships between variables and provide a framework for study design, a directed acyclic graph (DAG) was built using the *ggdag* package in R (Supplementary Figure S1). Our initial set of hypothesized confounders (based on previous studies and *a priori* knowledge) included marital status, maternal race, prenatal environmental tobacco smoke exposure, maternal age, maternal BMI, any hypertensive disorders during pregnancy, gestational diabetes, prenatal antibiotic use, child sex, parity, and birthweight z-score (4, 27–35). A combined approach to covariate adjustment was taken, which was informed upon by both knowledge-based strategies and statistical strategies (36). Specifically, among all variables indicated as potential adjustment covariates in the DAG, each was adjusted for individually to determine if the absolute percent change in the estimate was 10% or more, in which case it was included in the final set of adjustment covariates. Of the eleven candidate covariates, eight of them resulted in a 10% change or more (which included marital status, maternal race, maternal age, maternal BMI, any hypertensive disorders during pregnancy, gestational diabetes, child sex, and parity).

#### 2.4.3. hs-CRP class identification and modeling

Values of hs-CRP were log-transformed prior to analysis. To identify classes of hs-CRP trajectories, the R package *lcmm* (37) was used to fit growth mixture models (GMMs); additional details can be found in the online supplementary text. GMM results were compared to traditionally used cut points of hs-CRP of <1 mg/L, 1–3 mg/L, and >3 mg/L (21). However, due to small sample sizes at early time points, the top two categories were collapsed as ≥1 mg/L vs. <1 mg/L. The association between mode of delivery and hs-CRP (trajectory class or hs-CRP ≥ 1 mg/L) was evaluated using Poisson regression with robust error variances (38) to calculate risk ratios (RRs). Models were weighted by either the IPW described above, or each subject's posterior probability of being assigned to the specified hs-CRP class. For models using multiple imputed data, the *mianalyze* SAS procedure was used to pool Poisson regression estimates. Models were fit both unadjusted and adjusted for the previously described set of adjustment covariates. E-values were used to assess robustness to unmeasured confounding, which are defined as the minimum strength of association, on the risk ratio scale, that an unmeasured confounder would need to have with both the treatment and the outcome to fully explain away a specific treatment–outcome association, conditional on the measured covariates (39).

Because inflammatory responses may be race and sex dependent (40), effect modification for these two covariates was pre-specified and tested using interaction terms. Additionally, because women who deliver via C-section may be less likely to initiate or sustain breastfeeding (41), and breastfeeding has been associated with lower CRP levels in young adulthood (42), breastfeeding status at 1-month was a hypothesized mediator. BMI z-score at age 2 was also a hypothesized mediator, as C-section delivery results in an increased risk of childhood obesity (2), and obesity is a major determinant of CRP levels (13). Puberty status at age 10 was also tested for mediation, as mode of delivery may impact pubertal development (43) and pubertal status is associated with inflammatory markers (44). Further, the mediating effect of hs-CRP class on the association between mode of delivery and BMI z-score at age 10 was also examined. This same multiple imputed dataset was used for mediation models, which were fit using the R *mediation* package (45). Models were again weighted by posterior probability and adjusted for potential confounders. Statistical significance was set to 0.05 throughout analyses.

## 3. Results

### 3.1. Descriptive statistics

**Figure 1:**
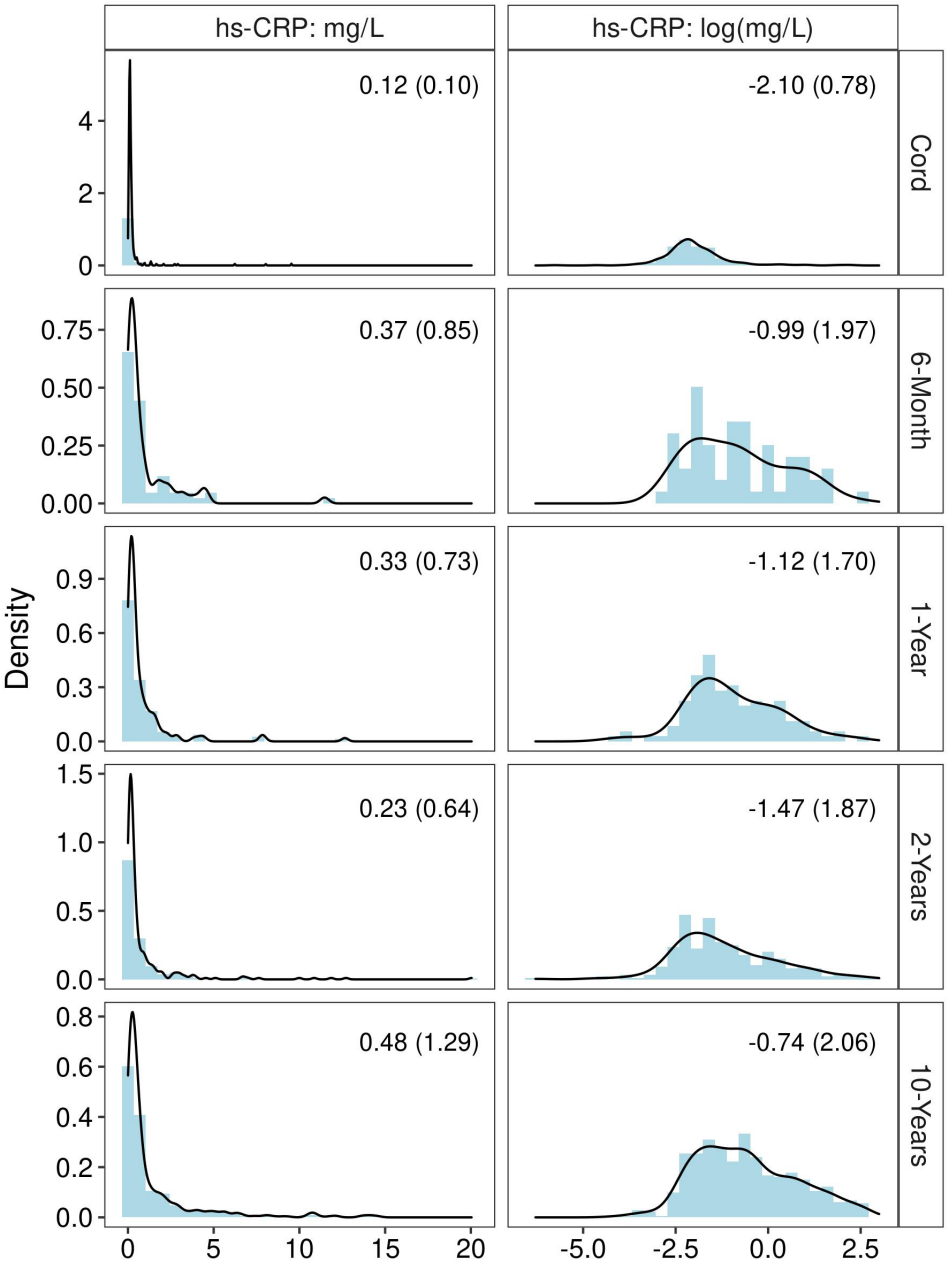
Distribution of hs-CRP before and after log transformation, at each time point. Statistic shown in the upper right corner of each panel is median (IQR).

Of the 564 children included in analyses (45% of the WHEALS birth cohort), the mean age at the 10-year visit was 10.3 years (SD = 0.9 years). Roughly 21% of females were in pre to early puberty at the 10-year visit, compared to 63% of males; 21% of children were obese at age 10. A total of 1,298 plasma samples across these 564 children were quantified for hs-CRP levels: 296 umbilical cord samples, 62 6-month samples, 111 1-year samples, 266 2-year samples, and 563 10-year samples. Values of hs-CRP (mg/L) among all samples ranged from 0.002 to 20.04 (median = 0.25, Q_1 _= 0.12, Q_3 _= 0.80). The median (IQR) at each time point is shown in Figure 1. The distribution was heavily right-skewed at each time point, but normalized through log transformation (Figure 1). A total of 10 (3.4%) of cord measurements were ≥1 mg/L, compared to 16 (25.8%) at 6-months, 26 (23.4%) at 1-year, 51 (19.2%) at 2-years, and 174 (30.9%) at 10-years. Though 17 children had 10-year hs-CRP > 10 mg/L, none met our definition of active infection by also having a white blood cell count >10 K/ul (blood samples collected at time of research visit rather than during a doctor's visit due to episode of illness). The majority of children had hs-CRP quantified at two time points [246 (44%)], followed by three time points [160 (28%)], one time point [104 (18%)], 4 time points [48 (9%)], and all 5 time points [6 (1%)].

A description of children who did and did not have hs-CRP levels quantified is shown in Supplementary Table S1. Prior to inverse probability weighting to account for selection bias, children who had at least one hs-CRP measurement had mothers who were more likely to report Health Alliance Plan (a health maintenance organization that is a subsidiary of Henry Ford Health) insurance coverage (*p* < 0.001), a Bachelor's degree or more (*p* < 0.001), and had higher household income levels (*p* < 0.001); they were also generally older (*p* < 0.001), were more likely to be married (*p* = 0.009), were less likely to smoke prenatally (*p* = 0.003), and were less likely to be exposed to environmental tobacco smoke prenatally (*p* = 0.036). Additionally, children who had hs-CRP measurements tended to weigh more at birth (*p* = 0.011). Though the rates of vaginal and C-section delivery did not differ between those who did and did not have at least one hs-CRP measurement (*p* = 0.861), the rate of missingness for specific delivery type was higher in those who did not have hs-CRP measurements (18.7% vs. 6.4%; *p* < 0.001). However, after inverse probability weighting, no covariates remained significant (all *p* ≥ 0.23). Additionally, though some covariates were highly imbalanced prior to weighting and greater than the threshold of 0.20 which indicates imbalance (max standardized difference = 0.63), this was also effectively mitigated after weighting (max standardized difference = 0.07).

### 3.2. hs-CRP latent classes

**Figure 2:**
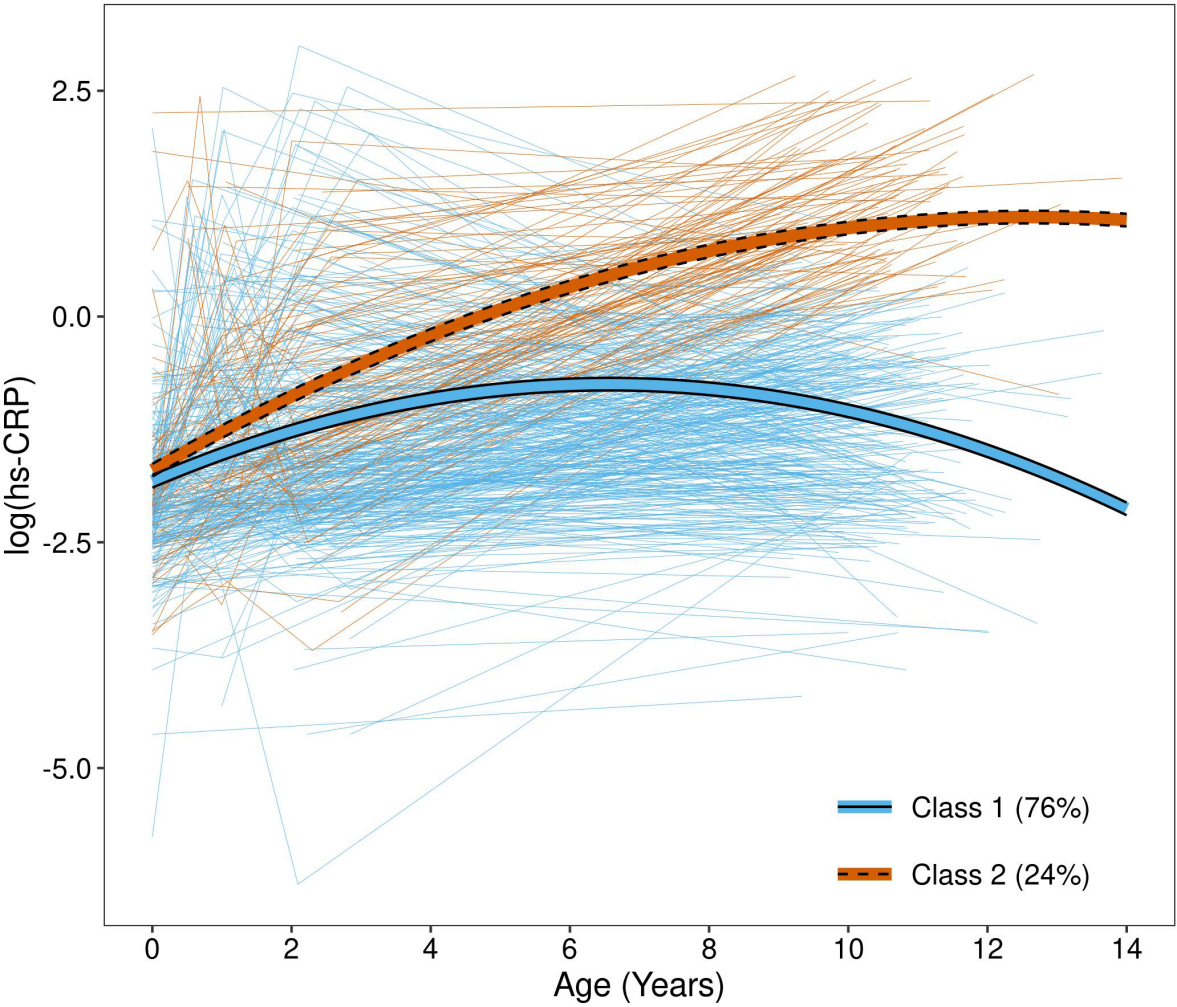
hs-CRP trajectories by class. Thick lines represent fitted mean trajectories from the growth mixture model, while thin, light lines represent observed measurements for each child. Trajectories are colored by class assignment, determined by maximum posterior probability.

When both model fit statistics (Supplementary Figure S2) and class interpretability (Supplementary Figure S3) were considered, the 2-class model was selected. A total of 430 (76%) children were assigned to class 1 while 134 (24%) were assigned to class 2 (Figure 2); the average maximum posterior probabilities were 0.91 and 0.78 in class 1 and 2, respectively. Class 1—which contained the majority of children—was characterized by low hs-CRP throughout childhood, which plateaued before 10 years of age. Class 2 was characterized by high and steadily increasing hs-CRP levels throughout childhood. When the association between hs-CRP class and hs-CRP ≥ 1 mg/L at each time point was examined, a significant association was found at age 10 years only. However, the effect size was very large, with 133 out of 134 children in hs-CRP class 2 having 10-year hs-CRP ≥ 1 mg/L (99.3%), compared to only 41 (9.6%) in hs-CRP class 1 (fisher's exact test *p* < 0.001). Children assigned to hs-CRP class 2 had mothers with higher prenatal BMI measurements, and they were more likely to have gestational diabetes and prenatal environmental tobacco smoke exposure during pregnancy (Table 1; all *p* < 0.05). Additionally, children assigned to class 2 had significantly higher BMI z-scores at age 2 (*p* = 0.005), but did not significantly differ on birthweight z-score (*p* = 0.94). Height, weight, and BMI z-score were all significantly higher in hs-CRP class 2, and these children were more likely to be obese at age 10 (all *p* < 0.001). Children in hs-CRP class 2 were less likely to be in pre to early puberty (*p* = 0.020), despite no differences in CRP class by sex (*p* = 0.69).

**Table 1:** Comparison of maternal and child characteristics by child hs-CRP class.

Covariate	Level	hs-CRP class		*p*-value^a^
		Class 1 *N* = 430	Class 2 *N* = 134	
		*N* (Column %) or *N*, Mean ± SD		
Mother married	No	152 (35.3%)	43 (32.1%)	0.49
	Yes	278 (64.7%)	91 (67.9%)	
Race-ethnicity of mother	White	105 (24.4%)	25 (18.7%)	0.060
	African American	271 (63%)	82 (61.2%)	
	Other/Mixed	54 (12.6%)	27 (20.1%)	
Maternal age at birth (years)	—	430, 30.2 ± 5.2	134, 29.9 ± 5.6	0.47
Maternal BMI at first prenatal visit (kg/m^2^)	—	419, 30.2 ± 8.3	128, 32.6 ± 7.7	0.004
Any hypertensive disorders during pregnancy	No	311 (88.6%)	92 (82.1%)	0.076
	Yes	40 (11.4%)	20 (17.9%)	
Gestational diabetes	No	329 (93.2%)	96 (85.7%)	0.014
	Yes	24 (6.8%)	16 (14.3%)	
Prenatal antibiotic use	No	172 (45.7%)	54 (47%)	0.82
	Yes	204 (54.3%)	61 (53%)	
Prenatal environmental tobacco smoke exposure	No	333 (77.4%)	92 (68.7%)	0.039
	Yes	97 (22.6%)	42 (31.3%)	
Birth weight z-score	—	407, −0.05 ± 1.02	124, −0.04 ± 1.07	0.94
Preterm Birth	No	392 (92.0%)	121 (93.1%)	0.69
	Yes	34 (8.0%)	9 (6.9%)	
Child sex	Male	217 (50.5%)	65 (48.5%)	0.69
	Female	213 (49.5%)	69 (51.5%)	
First born child	No	267 (62.1%)	78 (58.2%)	0.42
	Yes	163 (37.9%)	56 (41.8%)	
Breastfeeding status at 1-month	Formula Fed	73 (17.5%)	33 (25.8%)	0.080
	Mixed Feeding	285 (68.5%)	75 (58.6%)	
	Breastfeeding Only	58 (13.9%)	20 (15.6%)	
Puberty status at age 10	Pre - Early Puberty	188 (45.2%)	41 (32%)	0.020
	Mid Puberty	187 (45%)	75 (58.6%)	
	Late - Post Puberty	41 (9.9%)	12 (9.4%)	
Height at age 10 (cm)	—	427, 144.5 ± 9.1	134, 147.9 ± 9.3	<0.001
Weight at age 10 (kg)	—	427, 38.4 ± 10.8	134, 53.5 ± 18.7	<0.001
BMI z-score at age 2	—	323, 0.07 ± 1.06	105, 0.42 ± 1.28	0.005
BMI z-score at age 10	—	427, 0.12 ± 1.13	134, 1.32 ± 1.40	<0.001
Obese at age 10	No	388 (90.9%)	58 (43.3%)	<0.001
	Yes	39 (9.1%)	76 (56.7%)	

^a^ Calculated by ANOVA for numerical covariates and *χ*^2^ test for categorical covariates.

### 3.3. Association between mode of delivery and hs-CRP latent classes

When the association between mode of delivery and hs-CRP class was examined prior to covariate adjustment (Table 2), children delivered via C-section had a significantly higher risk of being in hs-CRP class 2 (the “high” hs-CRP class), relative to children delivered vaginally [Model 1; RR (95% CI) = 1.10 (1.02, 1.18); *p* = 0.016]. However, this association was diminished and no longer significant after using multiple imputation with covariate adjustment [Model 4; RR (95% CI) = 1.05 (0.95, 1.16); *p* = 0.29]. When C-section deliveries were further separated into planned and unplanned C-sections, an association was found among planned C-section deliveries, but not unplanned C-section deliveries, which persisted after covariate adjustment using multiple imputation estimates. Specifically, children delivered via planned C-section had 1.15 times higher risk of being in hs-CRP class 2, compared to vaginal deliveries [Model 4; RR 95% CI = 1.15 (1.01, 1.29); *p* = 0.028], while no association was found for unplanned C-section deliveries [Model 4; RR (95% CI) = 0.96 (0.84, 1.09); p = 0.49]. Results were very similar when the maximum posterior probability of class assignment was used as a subject weight rather than the IPW for analysis inclusion (Supplementary Table S2). E-value analysis indicated that the observed RR of 1.15 could be explained away by an unmeasured confounder that was associated with both planned C-section and hs-CRP class by a RR of 1.57, above and beyond the measured confounders, but weaker confounding could not do so; the CI could be moved to include the null by an unmeasured confounder that was associated with both planned C-section and hs-CRP class by a RR of 1.11 (beyond the measured confounders).

**Table 2:** Association between mode of delivery and hs-CRP class.

Mode of Delivery	*N* (%) in hs-CRP class 2	Model 1^a^		Model 2^b^		Model 3^c^		Model 4^d^	
		RR (95% CI)^e^	*p*-value	RR (95% CI)^e^	*p*-value	RR (95% CI)^e^	*p*-value	RR (95% CI)^e^	*p*-value
Vaginal	70 (19.9%)	1 [reference]		1 [reference]		1 [reference]		1 [reference]	
C-section (Any)^f^	62 (29.4%)	1.10 (1.02, 1.18)	0.016	1.01 (0.93, 1.09)	0.88	1.09 (0.99, 1.19)	0.075	1.05 (0.95, 1.16)	0.29
Planned C-section^g^	29 (35.8%)	1.16 (1.05, 1.27)	0.002	1.09 (0.98, 1.21)	0.13	1.17 (1.05, 1.31)	0.006	1.15 (1.01, 1.29)	0.028
Unplanned C-section^h^	22 (22.9%)	1.01 (0.93, 1.09)	0.88	0.92 (0.83, 1.01)	0.080	1.01 (0.90, 1.13)	0.90	0.96 (0.84, 1.09)	0.49

^a^ Weighted by inverse probability of inclusion + complete-case estimate + unadjusted.

^b^ Weighted by inverse probability of inclusion + complete-case estimate + adjusted for marital status, maternal race, maternal age, maternal BMI, any hypertensive disorders during pregnancy, gestational diabetes, child sex, and parity.

^c^ Weighted by inverse probability of inclusion + multiple imputation estimate + unadjusted.

^d^ Weighted by inverse probability of inclusion + multiple imputation estimate + adjusted for marital status, maternal race, maternal age, maternal BMI, any hypertensive disorders during pregnancy, gestational diabetes, child sex, and parity.

^e^ Risk ratios (RRs) representing the probability of being in hs-CRP class 2, comparing the specified mode of delivery to vaginal delivery.

^f^ Total *N* = 562, 446, 564, 564 in Models 1–4, respectively.

^g^ Total *N* = 432, 358, 460, 460 in Models 1–4, respectively.

^h^ Total *N* = 447, 372, 476, 476 in Models 1–4, respectively.

We did not find any evidence that the association between mode of delivery and hs-CRP class differed by maternal race (all interaction *p* ≥ 0.29) or child sex (all interaction *p* ≥ 0.90). Additionally, when the mediating effects of breastfeeding status at 1-month, BMI z-score at age 2, and puberty status at age 10 on the association between planned C-section delivery and hs-CRP class 2 were examined, no evidence of mediation was found (Supplementary Figure S4). For example, the average causal mediation effect (ACME) of BMI z-score at age 2—the estimated average increase in risk of hs-CRP class 2 that arrives as a result of BMI z-score at age 2 rather than ‘directly’ from planned C-section—was small and non-significant (increase in risk of 0.9%). On the other hand, the average direct effect (ADE)—the effect for planned C-section when BMI z-score at age 2 is held constant—was large and significant, where planned C-section resulted in approximately a 12% increase in the probability of hs-CRP class 2. The ADE therefore accounted for nearly all of the significant total effect. Results were very similar for breastfeeding status and puberty status.

### 3.4. Association between mode of delivery and hs-CRP ≥ 1 mg/L

When the association between mode of delivery and hs-CRP ≥ 1 mg/L at each time point was examined, many models at cord and 6-months did not converge due to small sample sizes (Supplementary Table S3). Though some associations were significant prior to covariate adjustment (models 1 and 3; 1-year and 10-years), adjusted models (2 and 4) did not demonstrate a significant association between mode of delivery and hs-CRP ≥ 1 mg/L. Though it did not reach statistical significance, children born via planned C-section had roughly 1.5 times higher risk of having a hs-CRP ≥ 1 mg/L at age 10 (RR (95% CI) = 1.51 (0.99, 2.32); *p* = 0.057), indicating a consistent direction of association compared to hs-CRP class.

### 3.5. Mediating effect of hs-CRP in mode of delivery/BMI at age 10 association

**Figure 3:**
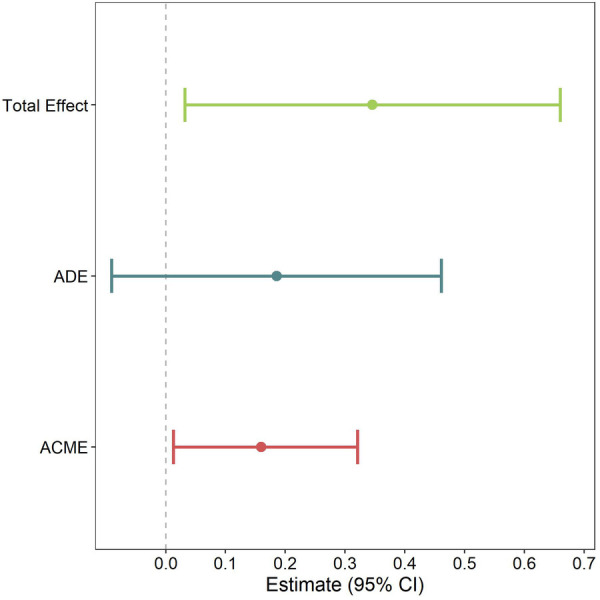
Model to examine whether CRP class mediates the association between planned C-section and BMI z-score at age 10. Pooled multiple imputation estimates are shown, which represent the mean difference in BMI z-score at age 10. Models are weighted by posterior probability and adjusted for marital status, maternal race, maternal age, maternal BMI, any hypertensive disorders during pregnancy, gestational diabetes, child sex, and parity. Average causal mediation effect (ACME); average direct effect (ADE).

We next performed mediation models to determine if hs-CRP class is a mediator in the planned C-section/BMI z-score at age 10 association we previously reported (4). We found that the effect of planned C-section on BMI z-score at age 10 was significantly mediated by hs-CRP class (Figure 3), with 43.4% of the association mediated by hs-CRP class. In addition to the mediated effect (the effect that arrives as a result of hs-CRP class rather than “directly” from planned C-section), the total effect was also significant. However, the direct effect (the effect of planned C-section on BMI z-score at age 10 when hs-CRP class is held constant) was non-significant. In other words, the effect of planned C-section on BMI z-score at age 10 seems to be largely driven by alterations in inflammation, as measured by hs-CRP class.

## 4. Discussion

In a racially and socioeconomically diverse US birth cohort, two hs-CRP trajectory classes from birth to age 10 were identified. The majority of children (76%) were assigned to a class that had low hs-CRP throughout childhood and plateaued before 10 years of age. The remaining 24% of children were assigned to a class which had high and steadily increasing hs-CRP throughout childhood, potentially indicative of chronic inflammation. Given that WHEALS is a socioeconomically and racially diverse birth cohort with a high rate of childhood obesity, we do not find the high rate of children exhibiting high hs-CRP levels to be surprising, as previous studies have shown racial disparities in CRP levels and associations with socioeconomic status (32), as well as associations with childhood obesity (46). When the association between mode of delivery and hs-CRP class was examined, children delivered via planned C-section were found to have a significantly higher risk of hs-CRP class 2 (high and increasing) relative to vaginal deliveries. Consistent with what we observed for the association between mode of delivery and pre-adolescent obesity (4), this association was not found among children delivered via unplanned C-section. Because partial labor is typically experienced during unplanned C-sections, these findings potentially suggest beneficial effects of partial labor. However, they could also reflect additional risk accompanying planned C-section, such as having a high-risk pregnancy or having a previous C-section delivery. Additionally, the effect was not significantly mediated by breastfeeding status at 1-month, BMI z-score at age 2, or puberty status at age 10. This suggests that the effect is not explained by planned C-section delivered babies having higher early childhood BMIs; they appear to be systemically inflamed throughout childhood independent of body size. At the same time, the association between planned C-section and BMI z-score age 10 was significantly mediated by hs-CRP class, indicating the effect may be through an inflammatory pathway. Other factors such as lipids/cholesterol, insulin resistance, and fasting glucose levels may also lie within the causal pathway as they have also been shown to relate to CRP levels (13); however, data on these biomarkers were not available.

Though planned C-section was significantly associated with an increased risk of the high and steadily increasing hs-CRP class, it did not significantly associate with hs-CRP ≥ 1 mg/L at each time point. The discrepancy in findings may be explained by a few reasons. Firstly, the growth mixture model takes a data-driven approach to determine what is considered “high” and “low” in this specific population of children by identifying homogeneous subgroups, as opposed to pre-specifying high or low cut points. The typically used cut points of hs-CRP analyzed here are based on tertiles of hs-CRP in an adult population, which were found to be predictive of cardiovascular disease risk more than twenty years ago (21). However, these cut points have not been validated in children to our knowledge. Secondly, because the growth mixture model examines longitudinal trends in hs-CRP, hs-CRP classes may be capturing more sustained and consistent inflammation over time, as opposed to a measurement at a single time point, which is subject to day-to-day variability.

Though hs-CRP has been linked to a wide range of chronic diseases in adults, few studies have examined the developmental origins of elevated CRP. A study examining the association between birth weight and CRP in early adulthood identified a non-linear relationship (42). This same study also found that breastfeeding was associated with lower CRP levels (42). A meta-analysis of 25 studies demonstrated a significant association between childhood trauma and inflammatory markers in adulthood, including CRP, IL-6, and TNF-α (47). Another study—which used cross-sectional U.S. National Health and Nutrition Examination Survey (NHANES) data—took a more holistic approach to examining a wide range of predictors in association with childhood hs-CRP levels; they found an inverse association between family income and hs-CRP levels, and that Mexican-American children had higher hs-CRP levels than both black and white children (34). Very few studies have examined the association between mode of delivery and childhood hs-CRP levels. In the US birth cohort Project Viva, adolescents born by C-section were shown to exhibit differences in certain metabolic health and inflammatory biomarkers, including lower adiponectin and increased insulin resistance (48). However, they did not find an association with CRP levels, even after examining planned and unplanned C-section separately. Unlike WHEALS where the majority of subjects were Black, the majority of subjects in Project Viva were White, which may explain differences in findings. A German birth cohort study examined cord blood hs-CRP levels and found a positive association with duration of labor—but not delivery mode—after covariate adjustment (49). However, they did not examine later hs-CRP measurements in childhood. In our analysis, the two trajectory classes were fairly similar at birth but later diverged; therefore, collection of later time points may be essential.

How C-section delivery impacts early life and childhood development is not well understood and requires additional research. However, there is strong and consistent evidence that C-section delivery results in a decreased colonization of several bacterial taxa in newborn stool samples that are normally acquired through vaginal delivery, perhaps most prominently, *Bacteroides* species (7). Alterations to the early life gut microbiome have been shown to impact inflammatory responses (50). A birth cohort that began in the Philippines in 1983 found that higher levels of microbial exposure in infancy (captured through proxy measures) were associated with lower hs-CRP production in adulthood (51). Though cross-sectional in design, a study of obese 10–16 year old children found that gut diversity and CRP levels were inversely correlated (52). Besides the so-called “hygiene hypothesis” and subsequent “microbial dysbiosis hypothesis”, other mechanisms have also been proposed. Labor and contractions (which are typically not experienced during planned C-section deliveries) may stimulate stress hormones and increase fetal blood flow, which prepare the infant to survive outside the womb (53). However, labor characteristics and other conditions associated with unplanned C-section were not systemically collected in this cohort. Epigenetic alterations may also play a role, as studies have demonstrated that these occur in stem cells following elective C-section delivery (54).

Our study is not without limitations. It is possible that some cases assigned to the high hs-CRP class have an active infection rather than chronic inflammation during sample collection. However, no children with hs-CRP > 10 mg/L at age 10 had a simultaneous white blood cell count >10 K/ul, and samples were collected during a research visit rather than during an episode of illness (and parents were asked to reschedule their visit if their child was ill). We chose not to exclude children with high hs-CRP alone because CRP > 10 mg/L has been shown to lack sensitivity in distinguishing acute from chronic inflammation (55), and removal of these children may exclude those at highest chronic disease risk (22). Further, given the large gap between sample collection at ages 2 and 10, we may be missing important windows of development.

Given the observational nature of a longitudinal birth cohort, unmeasured confounding (due to variables such as insulin resistance, lipids, or fasting glucose) as well as residual confounding is possible. Unmeasured confounding of the magnitude indicated by E-value analysis is certainly not implausible. However, E-values are interpreted as “above and beyond the measured confounders,” and given that our analysis has captured an extensive range of confounders that likely account for much of the confounding effects, the evidence for causality is moderately robust. Further, while most women who undergo emergency C-section experience at least some labor and most women with a planned C-section do not undergo any labor (56), there may be a small number of women where this relationship is not true and this may have introduced some exposure misclassification. Further, hs-CRP was only measured among children who had a plasma sample collected at the 10-year study visit (along with any earlier samples for those children) due to cost constraints, so sample size was reduced to roughly half of the cohort. With the observed sample size and assuming a 5% type I error rate, we are able to detect a RR of 1.8 for planned C-section vs. vaginal delivery with 80% power. Though our observed RR was smaller (RR = 1.15), we believe this is nevertheless a meaningful difference given the multifactorial causes of inflammation throughout early life. Additionally, selection bias due to loss to follow-up is possible; however, several SES-related factors such as education and birthweight were strongly associated with study inclusion, and this information was used for inverse probability weight calculation to attempt to account for this bias.

Despite these limitations, our study has several strengths, including the longitudinal design, where hs-CRP was measured at multiple time points from birth throughout childhood. Additionally, a racially and socioeconomically diverse birth cohort was leveraged, and these associations may be most relevant to black children, who have an increased burden of chronic diseases that typically develop earlier in adulthood compared to their white counterparts (57). Further, the latent class approach to profiling hs-CRP trajectories may be more insightful than standard mixed modeling and cross-sectional approaches.

Additional work is needed to understand the biological mechanisms behind the association between mode of delivery and hs-CRP, including whether microbiological differences underlie the association, and if the elevation of childhood hs-CRP due to planned C-section delivery translates to an increase in risk of chronic diseases later in life. Continued encouragement of vaginal delivery, in the absence of maternal and fetal indicators for C-section delivery, may promote future health of the offspring. Data from The Lacarus Randomized Controlled Trial, which is studying the induction of mild labor before elective C-section, may hold promise for reducing any longer-term negative sequala of C-section delivery in offspring (58). There is also a critical need for trials studying if “vaginal seeding” for the restoration of gut microbiota in C-section delivered infants reduces chronic disease risk (59).

## Data Availability

The raw data supporting the conclusions of this article will be made available by the authors, without undue reservation.
