# Supplementary material for: Progression of C-reactive protein from birth through preadolescence varies by mode of delivery
Source: Front Pediatr. 2023 Jun 14;11:1155852. doi: 10.3389/fped.2023.1155852 (PMC10304017; doi:10.3389/fped.2023.1155852)
Supplement: Supplementary file 1 [file Table1.docx]

**1 Extended Methods**

**1.1 Power**

With the observed sample size and assuming a 5% type I error rate, we are able to detect a risk ratio of 1.8 for planned C-section vs. vaginal delivery, and a RR of 1.75 for unplanned C-section vs. vaginal delivery with 80% power.

**1.2 Statistical Analysis**

Growth mixture models (GMMs) were fit to determine hs-CRP class using the unimputed data, which are estimated using full information maximum likelihood (FIML). Under FIML, data are assumed to be missing at random (MAR), which we believe is a reasonable assumption for partially missing plasma samples. Actual age of the child at each measurement was used rather than targeted time of collection. The best fitting shape of the trajectory was first determined by examining fit statistics under the 1-class model. Linear, quadratic, cubic, and various spline models were considered. Once the best-fitting trajectory shape was selected, the number of classes was selected by jointly evaluating model fit statistics, class interpretability, and sample size considerations. In order to ensure convergence to the global maximum of the log-likelihood, grid-searching was performed to efficiently explore the parameter space; the 1-class model was used to generate initial values.

**2 Extended Results**

**2.1 hs-CRP Latent Classes**

When various trajectory shapes were compared in the 1-class model, spline models were removed from consideration due to lack of convergence, leaving linear, quadratic, and cubic fits. Among these three, cubic obtained the best fit for AIC, while quadratic obtained the best fit for both BIC and size adjusted BIC; the quadratic model was selected to avoid overparameterization. Fit statistics of quadratic models with class sizes ranging from 1-4 are shown in Figure S1. Though the 3-class model obtained the minimum value for all three fit statistics, one class only contained 9 (1.6%) children and was therefore considered too small for analysis. Additionally, the mean trajectory line for this third class was “U-shaped”, but upon further inspection of these 9 individual-level trajectories, it appeared that only half of them followed this pattern well, while the other half simply had high hs-CRP levels (Figure S2). However, the 2-class model closely followed in terms of fit; in particular, the BICs were very similar (4321 vs. 4310 in the 2 vs. 3 class model). Balancing each of these considerations, the 2-class model was selected. The number of plasma samples did not differ by CRP class (Wilcoxon rank sum test p = 0.33),

**3 Supplementary Figures**


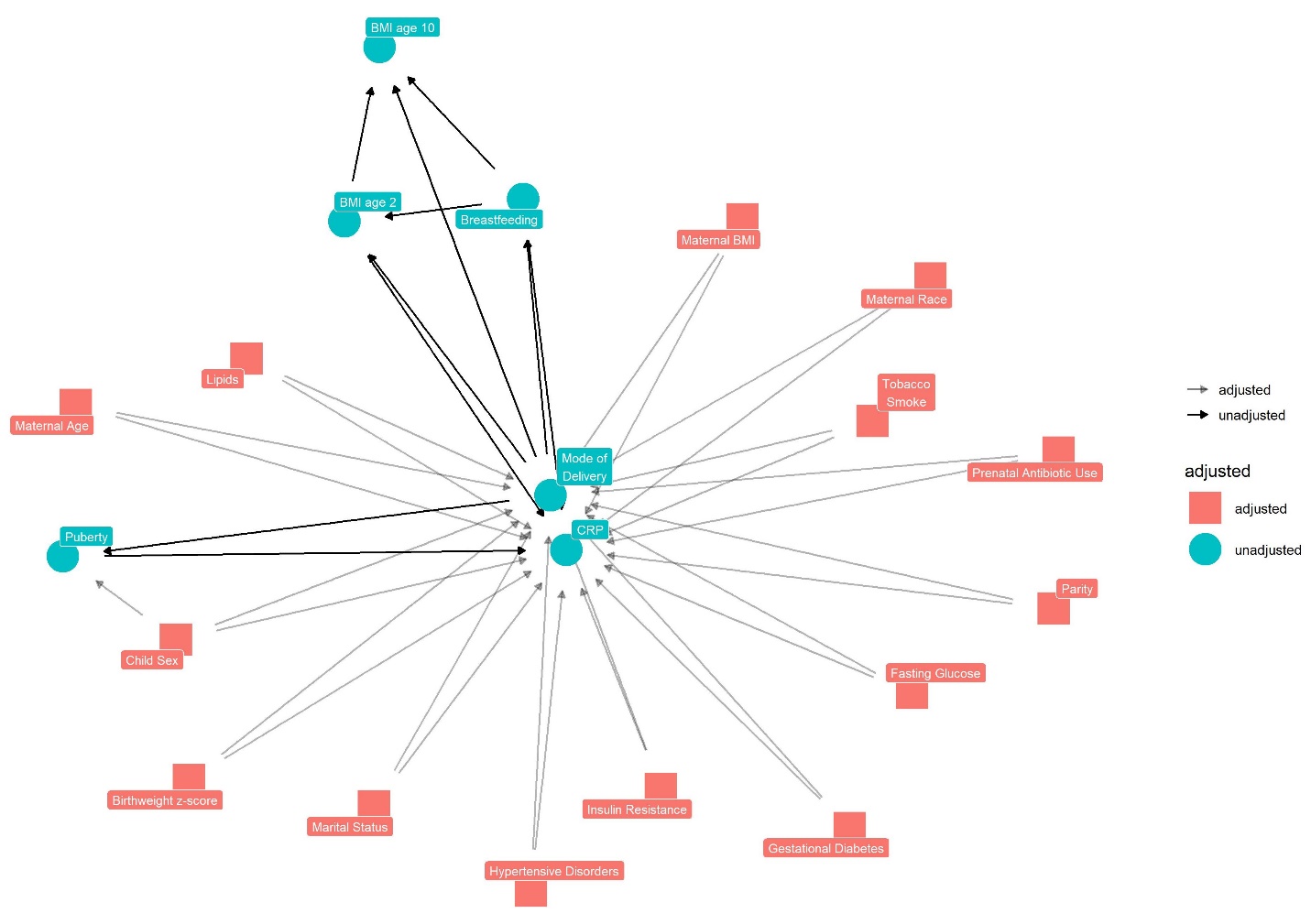


**Supplementary Figure 1.** Directed Acyclic Graph (DAG) as a conceptual framework for the association between mode of delivery and CRP throughout childhood. Potential adjustment set of covariates was calculated using the “ggdag” R package. Note that insulin resistance, lipids, and fasting glucose are unmeasured potential confounders.


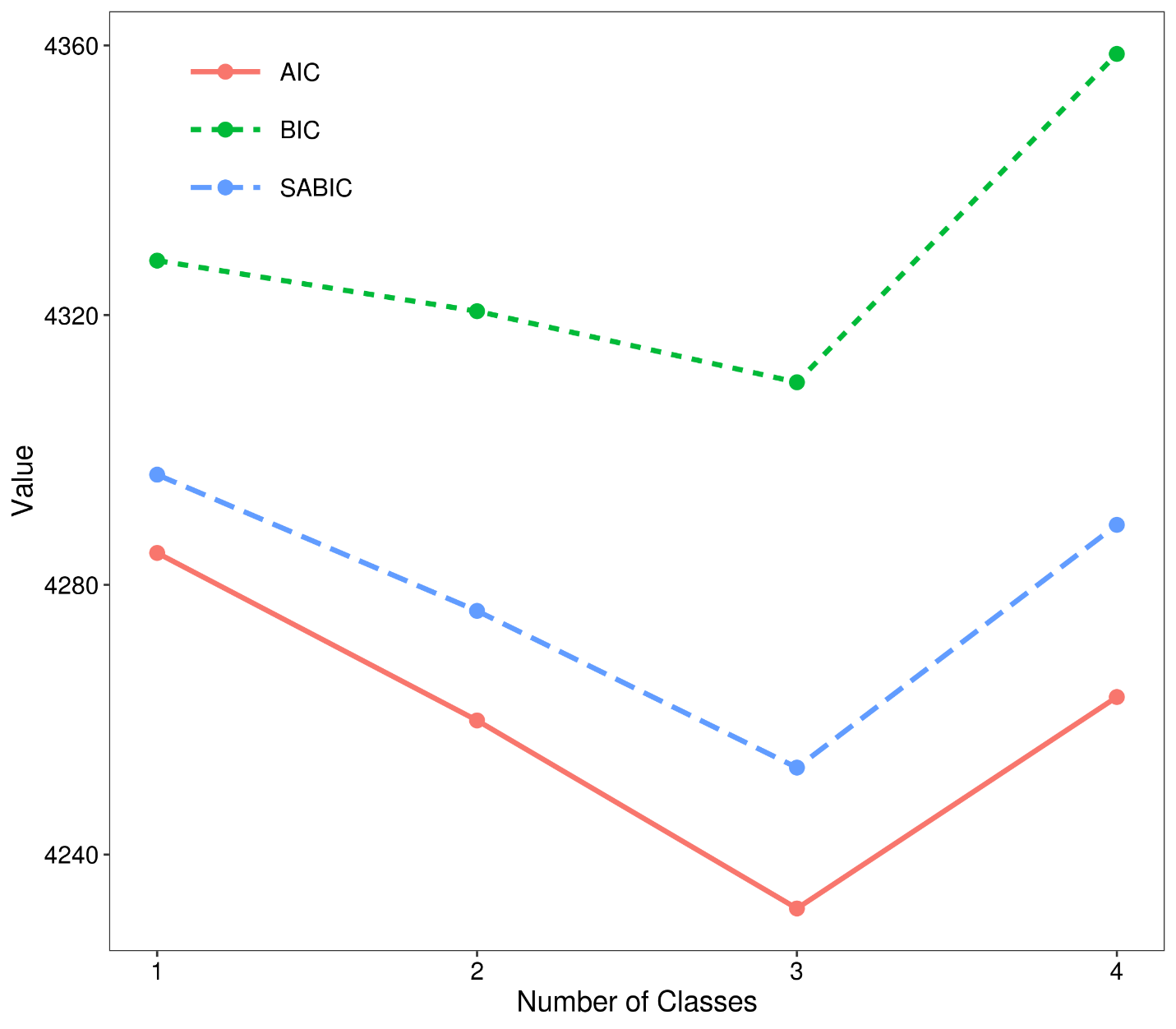


**Supplementary Figure 2**. Fit statistics for the quadratic model with class size ranging from 1 to 4.


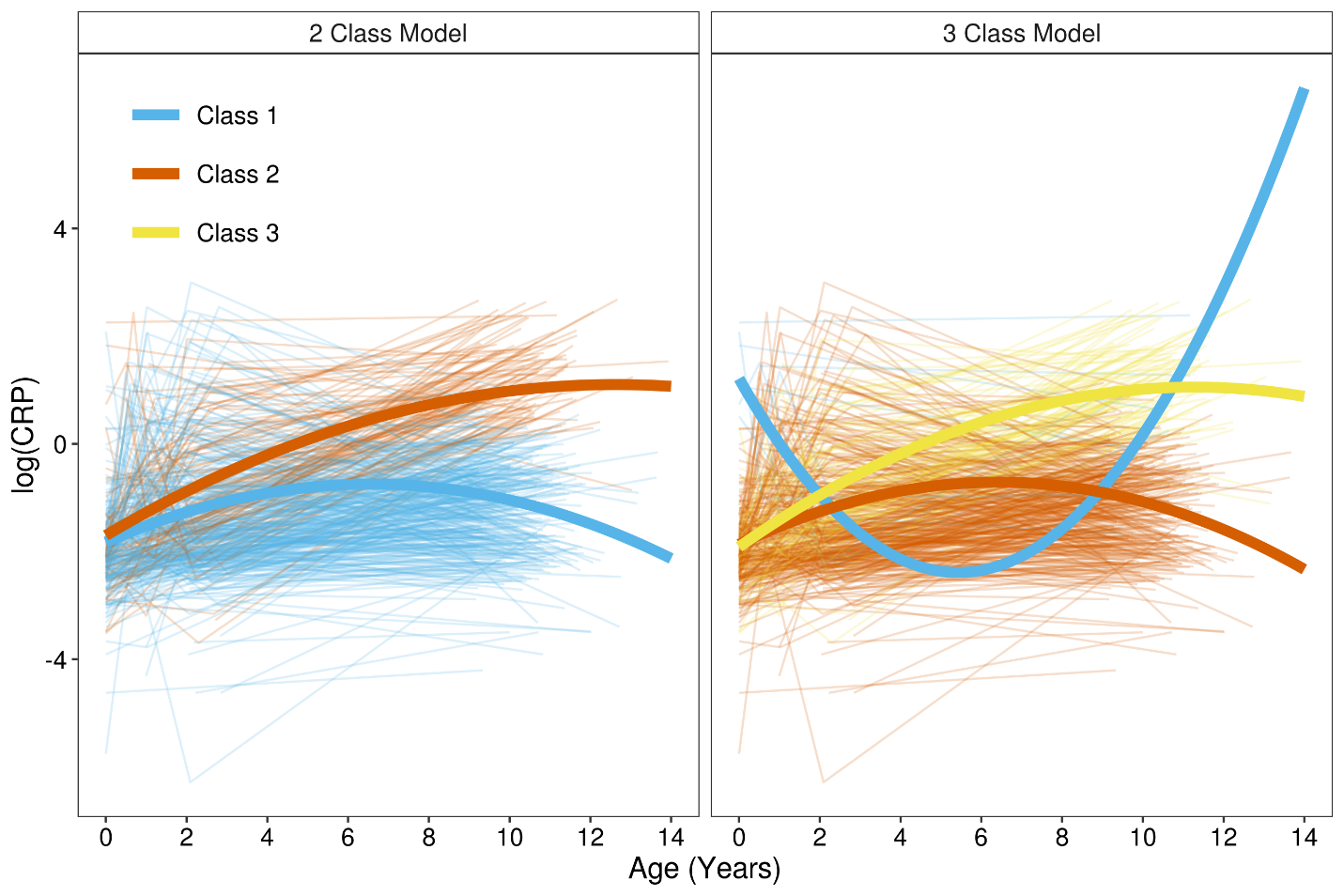


**Supplementary Figure 3.** Comparing the 2-class and 3-class solutions. The newly identified class in the 3-class solution (U-shaped class 1, in blue) contains 9 (1.6%) children. The thin blue lines seen behind it demonstrate a lack of fit. The majority of children assigned class 1 (blue) of the 2-class solution were assigned class 2 (red) of the 3-class solution (98%). Similarly, the majority of children assigned class 2 (red) of the 2-class solution were assigned class 3 (yellow) of the 3-class solution (94%).


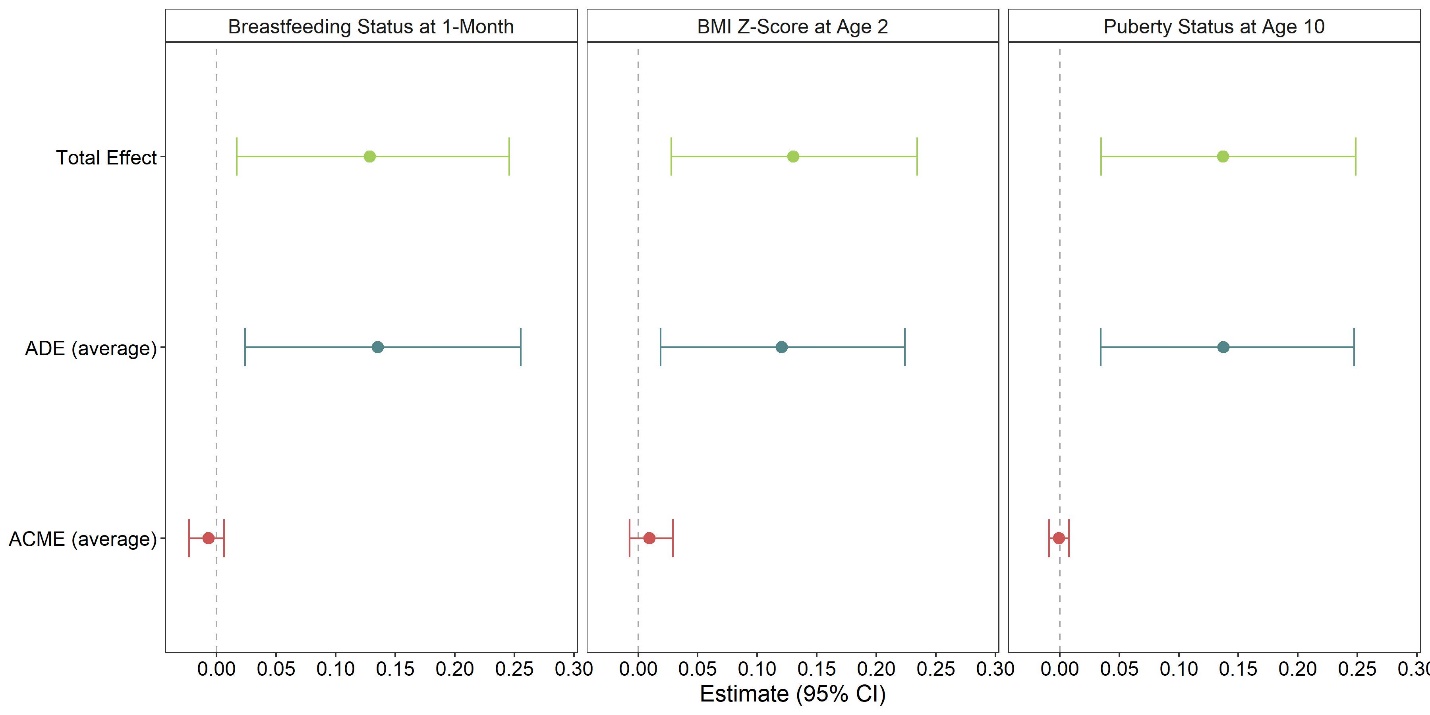


**Supplementary Figure 4.** Models to examine whether breastfeeding status at 1-month, BMI z-score at age 2, or puberty status at age 10 mediate the association between planned C-section and hs-CRP class 2. Pooled multiple imputation estimates are shown, which represent the increase in probability of hs-CRP class 2. Models are weighted by posterior probability and adjusted for marital status, maternal race, maternal age, maternal BMI, any hypertensive disorders during pregnancy, gestational diabetes, child sex, and parity. Abbreviations: average causal mediation effect (ACME); average direct effect (ADE).

**4 Supplementary Tables**

| **Supplementary Table 1. Comparison of children with and without hs-CRP measurements.** | | | | |  |  |  |
| --- | --- | --- | --- | --- | --- | --- | --- |
| **Covariate** | **Level** | **Has At Least One hs-CRP Measurement** | | **Before IPW^a^** | | **After IPW^a^** | |
|  |  | **No**  **N=694** | **Yes**  **N=564** |  |  |  |  |
|  |  | **N (Column %) or N, Mean±SD** | | **p-value^b^** | **D^c^** | **p-value^b^** | **D^c^** |
| Race-ethnicity of mother | White | 160 (23.1%) | 130 (23%) | 0.793 | 0.11 | 0.942 | 0.02 |
|  | African American | 425 (61.2%) | 353 (62.6%) |  |  |  |  |
|  | Other/Mixed | 109 (15.7%) | 81 (14.4%) |  |  |  |  |
| Insurance coverage | Health Alliance Plan | 213 (30.7%) | 289 (51.2%) | <0.001 | 0.63 | 0.967 | 0.03 |
|  | Other insurance | 220 (31.7%) | 211 (37.4%) |  |  |  |  |
|  | No insurance | 11 (1.6%) | 4 (0.7%) |  |  |  |  |
|  | Refused/Don't Know/Other Missing | 250 (36%) | 60 (10.6%) |  |  |  |  |
| Household income | <$20,000 | 117 (16.9%) | 65 (11.5%) | <0.001 | 0.26 | 0.946 | 0.07 |
|  | $20,000-<$40,000 | 167 (24.1%) | 128 (22.7%) |  |  |  |  |
|  | $40,000-<$80,000 | 198 (28.5%) | 149 (26.4%) |  |  |  |  |
|  | $80,000-<$100,000 | 56 (8.1%) | 79 (14%) |  |  |  |  |
|  | ≥$100,000 | 68 (9.8%) | 80 (14.2%) |  |  |  |  |
|  | Refused to Answer | 88 (12.7%) | 63 (11.2%) |  |  |  |  |
| Maternal education | <High school diploma | 55 (7.9%) | 19 (3.4%) | <0.001 | 0.37 | 0.936 | 0.02 |
|  | High school diploma | 142 (20.5%) | 86 (15.2%) |  |  |  |  |
|  | Some college | 345 (49.7%) | 260 (46.1%) |  |  |  |  |
|  | ≥Bachelor’s Degree | 152 (21.9%) | 199 (35.3%) |  |  |  |  |
| Mother married | No | 290 (41.8%) | 195 (34.6%) | 0.009 | 0.15 | 0.804 | 0.01 |
|  | Yes | 404 (58.2%) | 369 (65.4%) |  |  |  |  |
| Location of residence | Suburban | 292 (42.1%) | 263 (46.6%) | 0.106 | -0.09 | 0.624 | -0.02 |
|  | Urban | 402 (57.9%) | 301 (53.4%) |  |  |  |  |
| Maternal age at birth (years) | --- | 694, 29.1±5.2 | 564, 30.1±5.3 | <0.001 | 0.20 | 0.695 | -0.02 |
| Mom smoked during pregnancy | No | 594 (85.6%) | 514 (91.1%) | 0.003 | -0.17 | 0.554 | 0.02 |
|  | Yes | 100 (14.4%) | 50 (8.9%) |  |  |  |  |
| Prenatal environmental tobacco smoke exposure | No | 486 (70%) | 425 (75.4%) | 0.036 | -0.12 | 0.989 | -0.001 |
|  | Yes | 208 (30%) | 139 (24.6%) |  |  |  |  |
| Prenatal alcohol use | No | 669 (96.7%) | 537 (95.4%) | 0.239 | 0.17 | 0.638 | 0.05 |
|  | Yes | 23 (3.3%) | 26 (4.6%) |  |  |  |  |
| Prenatal indoor dogs | No | 539 (77.7%) | 416 (73.8%) | 0.107 | 0.09 | 0.946 | -0.003 |
|  | Yes | 155 (22.3%) | 148 (26.2%) |  |  |  |  |
| Prenatal indoor cats | No | 586 (84.4%) | 469 (83.2%) | 0.539 | 0.03 | 0.342 | 0.04 |
|  | Yes | 108 (15.6%) | 95 (16.8%) |  |  |  |  |
| Maternal doctor diagnosed hay fever or allergic rhinitis | No | 580 (84.3%) | 474 (85.7%) | 0.490 | -0.04 | 0.921 | -0.01 |
|  | Yes | 108 (15.7%) | 79 (14.3%) |  |  |  |  |
| Maternal doctor diagnosed asthma | No | 557 (80.3%) | 448 (79.6%) | 0.763 | 0.02 | 0.745 | -0.01 |
|  | Yes | 137 (19.7%) | 115 (20.4%) |  |  |  |  |
| Mode of delivery^d^ | Vaginal | 433 (62.9%) | 351 (62.5%) | 0.861 | 0.01 | 0.729 | 0.01 |
|  | C-Section | 255 (37.1%) | 211 (37.5%) |  |  |  |  |
| Mode of delivery (specific type) | Unknown | 130 (18.7%) | 36 (6.4%) | <0.001 | 0.43 | 0.687 | 0.06 |
|  | Vaginal | 433 (62.4%) | 351 (62.2%) |  |  |  |  |
|  | Planned C-Section | 66 (9.5%) | 81 (14.4%) |  |  |  |  |
|  | Unplanned C-Section | 65 (9.4%) | 96 (17%) |  |  |  |  |
| First born child | No | 453 (65.3%) | 345 (61.2%) | 0.133 | 0.09 | 0.487 | 0.03 |
|  | Yes | 241 (34.7%) | 219 (38.8%) |  |  |  |  |
| Child sex | Male | 340 (49.1%) | 282 (50%) | 0.741 | -0.02 | 0.232 | 0.05 |
|  | Female | 353 (50.9%) | 282 (50%) |  |  |  |  |
| Gestational age at delivery (weeks) | --- | 676, 38.7±1.8 | 556, 38.8±1.6 | 0.173 | 0.08 | 0.901 | -0.005 |
| Birth Weight (grams) | --- | 647, 3265±559 | 535, 3351±589 | 0.011 | 0.14 | 0.614 | -0.02 |
| ^a^*Inverse probability weighting (IPW) to account for selection bias.* | | | | | | | |
| ^b^*Calculated by ANOVA for numerical covariates and chi-square test for categorical covariates.* | | | | | | | |
| ^c^*Standardized difference, defined as the difference in means or proportions divided by standard error.* | | | | | | | |
| ^d^*Not included in inverse probability weighting, but specific mode of delivery type was included (including both would result in zero cells).* | | | | | | | |

| **Supplementary Table 2. Association between mode of delivery and hs-CRP class, using maximum posterior probability as subject weight rather than inversion probability of inclusion.** | | | | | | | | | |
| --- | --- | --- | --- | --- | --- | --- | --- | --- | --- |
| **Mode of Delivery** | **N (%) in hs-CRP Class 2** | **Model 1^a^** | | **Model 2^b^** | | **Model 3^c^** | | **Model 4^d^** | |
|  |  | **RR (95% CI)^e^** | **p-value** | **RR (95% CI)^e^** | **p-value** | **RR (95% CI)^e^** | **p-value** | **RR (95% CI)^e^** | **p-value** |
| Vaginal | 70 (19.9%) | 1 [reference] |  | 1 [reference] |  | 1 [reference] |  | 1 [reference] |  |
| C-section (Any)^f^ | 62 (29.4%) | 1.07 (1.01, 1.13) | 0.017 | 1.01 (0.94, 1.08) | 0.89 | 1.07 (1.01, 1.14) | 0.035 | 1.04 (0.97, 1.11) | 0.30 |
| Planned C-section^g^ | 29 (35.8%) | 1.13 (1.04, 1.23) | 0.005 | 1.08 (0.98, 1.18) | 0.14 | 1.13 (1.04, 1.23) | 0.004 | 1.10 (1.01, 1.21) | 0.029 |
| Unplanned C-section^h^ | 22 (22.9%) | 1.02 (0.95, 1.10) | 0.62 | 0.94 (0.86, 1.02) | 0.12 | 1.02 (0.94, 1.09) | 0.69 | 0.97 (0.89, 1.05) | 0.45 |
| ^a^*Weighted by inverse probability of inclusion + complete-case estimate + unadjusted.* | | | | | | | | | |
| ^b^*Weighted by inverse probability of inclusion + complete-case estimate + adjusted for marital status, maternal race, maternal age, maternal BMI, any hypertensive disorders during pregnancy, gestational diabetes, child sex, and parity.* | | | | | | | | | |
| ^c^*Weighted by inverse probability of inclusion + multiple imputation estimate + unadjusted.* | | | | | | | | | |
| ^d^*Weighted by inverse probability of inclusion + multiple imputation estimate + adjusted for marital status, maternal race, maternal age, maternal BMI, any hypertensive disorders during pregnancy, gestational diabetes, child sex, and parity.* | | | | | | | | | |
| ^e^*Risk ratios (RRs) representing the probability of being in hs-CRP class 2, comparing the specified mode of delivery to vaginal delivery.* | | | | | | | | | |
| ^f^*Total N=562, 446, 564, 564 in Models 1-4, respectively.* | | | | | | | | | |
| ^g^*Total N=432, 358, 460, 460 in Models 1-4, respectively.* | | | | | | | | | |
| ^h^*Total N=447, 372, 476, 476 in Models 1-4, respectively.* | | | | | | | | | |

| **Supplementary Table 3. Association between mode of delivery and hs-CRP****≥1 mg/L at each time point.** | | | | | | | | | |
| --- | --- | --- | --- | --- | --- | --- | --- | --- | --- |
| **Time of CRP Measurement** | **Mode of Delivery** | **Model 1^a^** | | **Model 2^b^** | | **Model 3^c^** | | **Model 4^d^** | |
|  |  | **RR (95% CI)^e^** | **p-value** | **RR (95% CI)^e^** | **p-value** | **RR (95% CI)^e^** | **p-value** | **RR (95% CI)^e^** | **p-value** |
| Cord | C-section (Any) | 0.54 (0.12, 2.32) | 0.40 | N/A^f^ | N/A | 0.93 (0.22, 3.86) | 0.92 | N/A | N/A |
|  | Planned C-section | 0.60 (0.07, 5.02) | 0.64 | N/A | N/A | 0.69 (0.08, 5.70) | 0.73 | N/A | N/A |
|  | Unplanned C-section | 0.77 (0.15, 3.96) | 0.76 | N/A | N/A | 1.04 (0.20, 5.28) | 0.96 | N/A | N/A |
| 6 Months | C-section (Any) | 1.20 (0.46, 3.13) | 0.72 | 1.40 (0.31, 6.27) | 0.66 | 0.86 (0.40, 1.87) | 0.71 | 0.78 (0.34, 1.75) | 0.53 |
|  | Planned C-section | 0.86 (0.15, 4.97) | 0.86 | N/A | N/A | 0.52 (0.15, 1.88) | 0.32 | 0.45 (0.12, 1.65) | 0.22 |
|  | Unplanned C-section | 1.77 (0.66, 4.75) | 0.26 | 1.39 (0.39, 4.91) | 0.61 | 1.14 (0.53, 2.43) | 0.73 | 1.07 (0.49, 2.35) | 0.86 |
| 1 Year | C-section (Any) | 0.29 (0.11, 0.75) | 0.011 | 0.44 (0.14, 1.37) | 0.16 | 0.73 (0.40, 1.36) | 0.32 | 0.72 (0.40, 1.30) | 0.27 |
|  | Planned C-section | 0.35 (0.09, 1.39) | 0.13 | 0.23 (0.05, 1.09) | 0.064 | 0.69 (0.31, 1.54) | 0.36 | 0.63 (0.29, 1.40) | 0.25 |
|  | Unplanned C-section | 0.41 (0.13, 1.25) | 0.12 | 0.57 (0.13, 2.45) | 0.45 | 0.75 (0.33, 1.73) | 0.50 | 0.78 (0.34, 1.76) | 0.54 |
| 2 Years | C-section (Any) | 0.95 (0.56, 1.63) | 0.86 | 1.07 (0.61, 1.87) | 0.82 | 1.08 (0.64, 1.83) | 0.77 | 1.14 (0.66, 1.95) | 0.64 |
|  | Planned C-section | 0.77 (0.35, 1.68) | 0.52 | 0.78 (0.34, 1.78) | 0.55 | 0.96 (0.46, 2.01) | 0.92 | 0.94 (0.47, 1.87) | 0.85 |
|  | Unplanned C-section | 1.08 (0.59, 1.98) | 0.79 | 1.33 (0.69, 2.56) | 0.40 | 1.17 (0.61, 2.23) | 0.64 | 1.32 (0.67, 2.59) | 0.42 |
| 10 Years | C-section (Any) | 1.34 (0.99, 1.81) | 0.053 | 1.03 (0.74, 1.43) | 0.85 | 1.35 (0.95, 1.90) | 0.090 | 1.20 (0.81, 1.77) | 0.35 |
|  | Planned C-section | 1.62 (1.18, 2.23) | 0.003 | 1.34 (0.92, 1.96) | 0.13 | 1.68 (1.15, 2.45) | 0.008 | 1.51 (0.99, 2.32) | 0.057 |
|  | Unplanned C-section | 1.07 (0.74, 1.55) | 0.71 | 0.73 (0.46, 1.15) | 0.18 | 1.05 (0.65, 1.69) | 0.84 | 0.88 (0.51, 1.53) | 0.66 |
| ^a^*Weighted by inverse probability of inclusion + complete-case estimate + unadjusted.* | | | | | | | | | |
| ^b^*Weighted by inverse probability of inclusion + complete-case estimate + adjusted for marital status, maternal race, maternal age, maternal BMI, any hypertensive disorders during pregnancy, gestational diabetes, child sex, and parity.* | | | | | | | | | |
| ^c^*Weighted by inverse probability of inclusion + multiple imputation estimate + unadjusted.* | | | | | | | | | |
| ^d^*Weighted by inverse probability of inclusion + multiple imputation estimate + adjusted for marital status, maternal race, maternal age, maternal BMI, any hypertensive disorders during pregnancy, gestational diabetes, child sex, and parity.* | | | | | | | | | |
| ^e^*Risk ratios (RRs) representing the probability of hs-CRP≥1 mg/L, comparing the specified mode of delivery to vaginal delivery.* | | | | | | | | | |
| ^f^*Model did not converge.* | | | | | | | | | |
